# Chemokine CCL4 Induced in Mouse Brain Has a Protective Role against Methylmercury Toxicity

**DOI:** 10.3390/toxics6030036

**Published:** 2018-07-07

**Authors:** Tsutomu Takahashi, Min-Seok Kim, Miyuki Iwai-Shimada, Masatake Fujimura, Takashi Toyama, Akira Naganuma, Gi-Wook Hwang

**Affiliations:** 1 Laboratory of Molecular and Biochemical Toxicology, Graduate School of Pharmaceutical Sciences, Tohoku University, Aoba-ku, Sendai 980-8578, Japan; tsutomu@toyaku.ac.jp (Ts.T.); assams7@naver.com (M.-S.K.); iwai.miyuki@nies.go.jp (M.I.-S.); takashi.toyama.c6@tohoku.ac.jp (Ta.T.); naganuma@m.tohoku.ac.jp (A.N.); 2 Department of Environmental Health, School of Pharmacy, Tokyo University of Pharmacy and Life Sciences, 1432-1 Horinouchi, Hachioji, Tokyo 192-0392, Japan; 3 Department of Inhalation Toxicology Research, Korea Institute of Toxicology, Jeonbuk 56212, Korea; 4 Center for Health and Environmental Risk Research, National Institute for Environmental Studies, Onogawa 16-2, Tsukuba, Ibaraki 305-8506, Japan; 5 Department of Basic Medical Science, National Institute for Minamata Disease, Kumamoto 867-0008, Japan; fujimura@nimd.go.jp

**Keywords:** methylmercury, brain, chemokine, CCL4

## Abstract

Methylmercury (MeHg) is selectively toxic to the central nervous system, but mechanisms related to its toxicity are poorly understood. In the present study, we identified the chemokine, C-C motif Chemokine Ligand 4 (CCL4), to be selectively upregulated in the brain of MeHg-administered mice. We then investigated the relationship between CCL4 expression and MeHg toxicity using in vivo and in vitro approaches. We confirmed that in C17.2 cells (a mouse neural stem cell line) and the mouse brain, induction of CCL4 expression occurs prior to cytotoxicity caused by MeHg. We also show that the addition of recombinant CCL4 to the culture medium of mouse primary neurons attenuated MeHg toxicity, while knockdown of CCL4 in C17.2 cells resulted in higher MeHg sensitivity compared with control cells. These results suggest that CCL4 is a protective factor against MeHg toxicity and that induction of CCL4 expression is not a result of cytotoxicity by MeHg but is a protective response against MeHg exposure.

## 1. Introduction

Methylmercury (MeHg) is an environmental pollutant well known to cause Minamata disease [1]. MeHg causes central nervous system (CNS) disorders whose main symptoms include sensory paralysis, speech disorder, ataxia, and visual field narrowing [2,3]. MeHg can easily pass through the blood-placental barrier and can therefore affect the brain of the immature fetus [4,5]. Although MeHg is selectively toxic in the brain, mechanisms related to this selectivity and defense mechanisms are still unknown. We have analyzed patterns of gene expression in the brains of mice that were administered MeHg and have identified a number of upregulated genes [6,7,8]. Among these genes, many encode cytokines, such as chemokines and interleukins. One such gene is C-C motif Chemokine Ligand 4 (CCL4), which is specifically upregulated in the brain by MeHg [9]. CCL4, also called macrophage inflammatory protein 1β (MIP1β), was identified as an inflammatory protein produced from macrophages [10]. CCL4, secreted extracellularly, binds to chemokine receptors (CCR1, CCR5) and is involved in leukocyte infiltration and activation [11]. CCL4 is secreted from glial cells and astrocytes in the CNS and has been suggested to be involved in the progression of various brain diseases, including Alzheimer’s disease, multiple sclerosis, and ischemic brain disease [12,13,14,15]. Nevertheless, the functions of CCL4 in the brain are unclear and the relationship between chemokines and MeHg toxicity is poorly understood. Recently, Godefroy et al. reported that CCL2, another C-C chemokine, attenuates MeHg toxicity in rat primary neuron cultures [16], but the mechanism for this is not understood. In this study, we investigated the relationship between CCL4 expression and MeHg toxicity, in vivo and in vitro, using mice and neuronal cell lines.

## 2. Materials and Methods

### 2.1. Animal Experiments

Eight-week-old male C57BL/6 mice were purchased from Japan SLC, Inc. (Shizuoka, Japan). The mice were housed in plastic cages (five animals per cage) at 22 ± 2 °C with a relative humidity of 55 ± 20% under a 12-h light-dark cycle and allowed free access to chow (F-2, Oriental Yeast, Tokyo, Japan) and water. All experiments were performed in accordance with the Regulations for Animal Experiments and Related Activities at Tohoku University, and were approved by the Animal Care Committee of Tohoku University (No.: 2016PhA-001, Date: 8 February 2016). After an adaptation period, mice were randomly divided into control (*n* = 5) and MeHg-treated (*n* = 5) groups. Methylmercuric chloride (25 mg/kg), dissolved in physiological saline, was administered by subcutaneous injection. After the indicated time period, the mice were dissected and each organ was subjected to the various assays.

### 2.2. Immunochemistry

Immunohistochemistry was performed as described previously [17,18,19]. Paraffin embedded sections were cut using a microtome, and immunohistochemistry was performed using the Vectastain Elite ABC Kit (Vector Laboratories, Burlingame, CA, USA) with an antibody to neuronal nuclei (NeuN) (Chemicon, Temecula, CA, USA).

### 2.3. Cell Culture

Mouse C17.2 neural stem cells were cultured in Dulbecco’s modified Eagle’s medium (DMEM) (Nissui Pharmaceutical, Tokyo, Japan) supplemented with 10% heat-inactivated fetal bovine serum (FBS), 2 mM/L l-glutamine, and antibiotic (100 IU/mL penicillin and 100 mg/mL streptomycin) in a humidified 5% CO_2_ atmosphere at 37 °C. Mouse primary cerebellar granule cells were cultured in Neurobasal-A medium (Thermo Fisher Scientific, Waltham, MA, USA) containing 2% B25 (Thermo Fisher Scientific), 1% FBS, and 25 mM KCl in 12-well plates for 2 weeks.

### 2.4. siRNA Transfection

Double-stranded siRNA for CCL4 (target sequence: CTTTGTGATGGATTACTATTT) and negative control siRNA were purchased from Sigma-Aldrich (St. Louis, MO, USA). C17.2 cells were transfected with siRNAs using HiPerFect transfection reagent (Qiagen, Germantown, MD, USA) according to the manufacturer’s protocol.

### 2.5. Cell Viability Assay

C17.2 cells and mouse primary cerebellar granule cells were cultured in media containing methylmercuric chloride for 24 h. Cell viability was measured using the alamarBlue^®^ assay (Biosource, Camarillo, CA, USA). Fluorescence was measured using a Gemini XPS microplate spectrofluorometer (Molecular Devices, Sunnyvale, CA, USA) (excitation wavelength 545 nm; emission wavelength 590 nm). Trypan blue assays were performed using a Vi-Cell XR cell viability analyzer (Beckman coulter, San Diego, CA, USA).

### 2.6. Measurement of CCL4 mRNA Levels by Quantitative Real-Time PCR

Total RNA from organs and cells was isolated using the Isogen II Kit (Nippon Gene, Tokyo, Japan) according to the manufacturer’s protocol. The first-strand cDNA was synthesized from 500 ng of total RNA using the PrimeScriptTM RT Reagent Kit (Takara, Shiga, Japan). Quantitative real-time PCR analysis was performed using SYBR Premix EX Taq (Takara) with a Thermal Cycler Dice^®^ (Takara). The PCR primers used included the following: CCL4, 5′-ACCCTGTGACATTTCACGGAG-3′ (sense) and 5′-GTACTCGATTGATAGAGGAC-3′ (antisense); and GAPDH, 5′-ATCACCATCTTCCAGGAGCGA-3′ (sense) and 5′-AGGGGCCATCCACAGTCTT-3′ (antisense). Fold changes in mRNA levels were determined from standard curves after calibration of the assay. CCL4 mRNA levels were normalized to those of GAPDH.

### 2.7. Statistical Analysis

If not stated otherwise, statistical significance of the data was determined using analysis of variance (ANOVA) with Dunnett’s post hoc test.

## 3. Results

### 3.1. CCL4 Expression Is Induced Prior to Neuronal Damage Caused by MeHg

**Figure 1:**
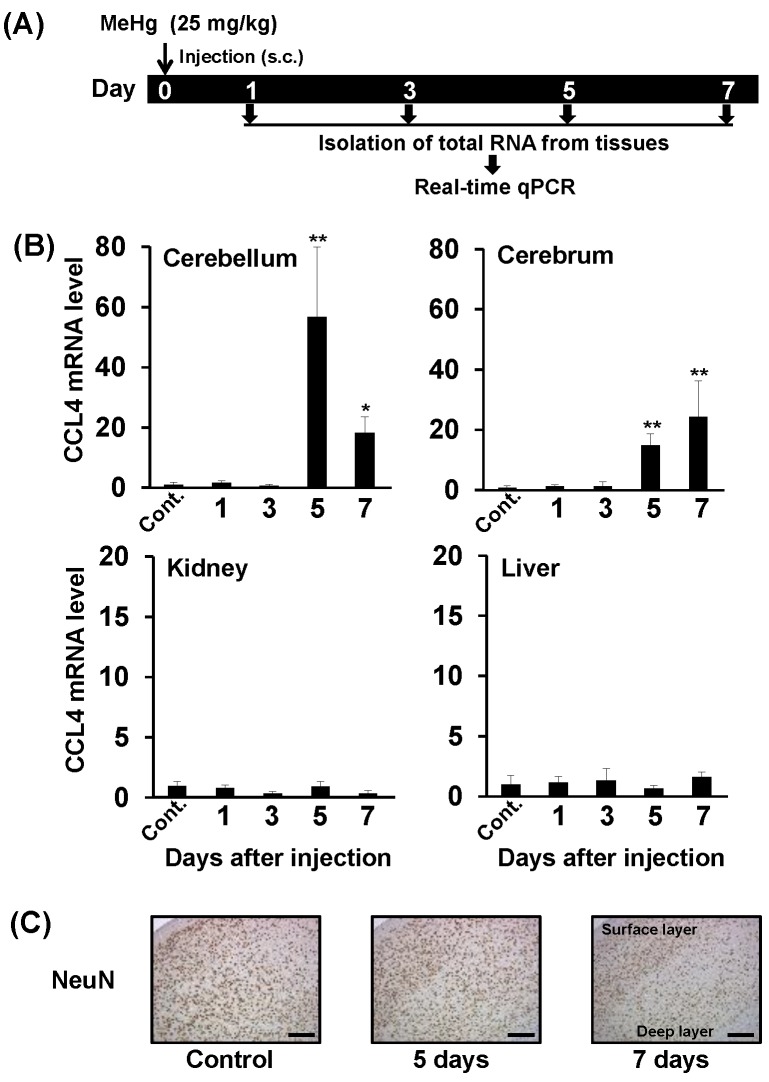
Relationship between neurological damage and CCL4 expression in the brains of mice treated with methylmercury. C57BL/6 mice were injected once subcutaneously with methylmercuric chloride (MeHg) (25 mg/kg). (**A**) Selected organs (cerebellum, cerebrum, liver and kidney) were dissected 1, 3, 5, or 7 days after the injection. Cont.: Control. (**B**) CCL4 mRNA levels in each organ were measured by quantitative real-time PCR. Data are represented as mean ± SD, * *p* < 0.05; ** *p* < 0.01 compared with “Control”. (**C**) NeuN (neuron marker)-positive cells in the cerebral cortex detected by immunostaining. Scale bars represent 250 μm.

To study the relationship between MeHg toxicity and CCL4 expression in mice, we administered a single dose of methylmercuric chloride (25 mg/kg) and then used real-time qPCR to investigate changes in CCL4 mRNA levels in cerebrum, cerebellum, kidney, and liver over time (Figure 1A). CCL4 mRNA levels were elevated in the cerebrum and cerebellum from 5 days after MeHg administration (Figure 1B). CCL4 expression was not induced in the kidney or liver at any time point tested (Figure 1B). This indicates that MeHg induces CCL4 expression in a brain-specific manner. We then investigated pathological changes in the brains of mice after single-dose administration of 25 mg/kg methylmercuric chloride. As a reference index, we counted cells that were positive for the neuronal marker, NeuN. We observed almost no change in NeuN-positive cell numbers in the cerebellum, even at 7 days after MeHg administration (data not shown). In the cerebrum, no changes were found in the number of NeuN-positive cells up to 5 days, and on day 7 a slight decrease in the number of NeuN-positive cells was observed (Figure 1C). Therefore, CCL4 expression was induced in the mouse brain prior to neuronal damage caused by MeHg.

### 3.2. CCL4 Attenuates MeHg Toxicity in Primary Mouse Neuron Cultures

**Figure 2:**
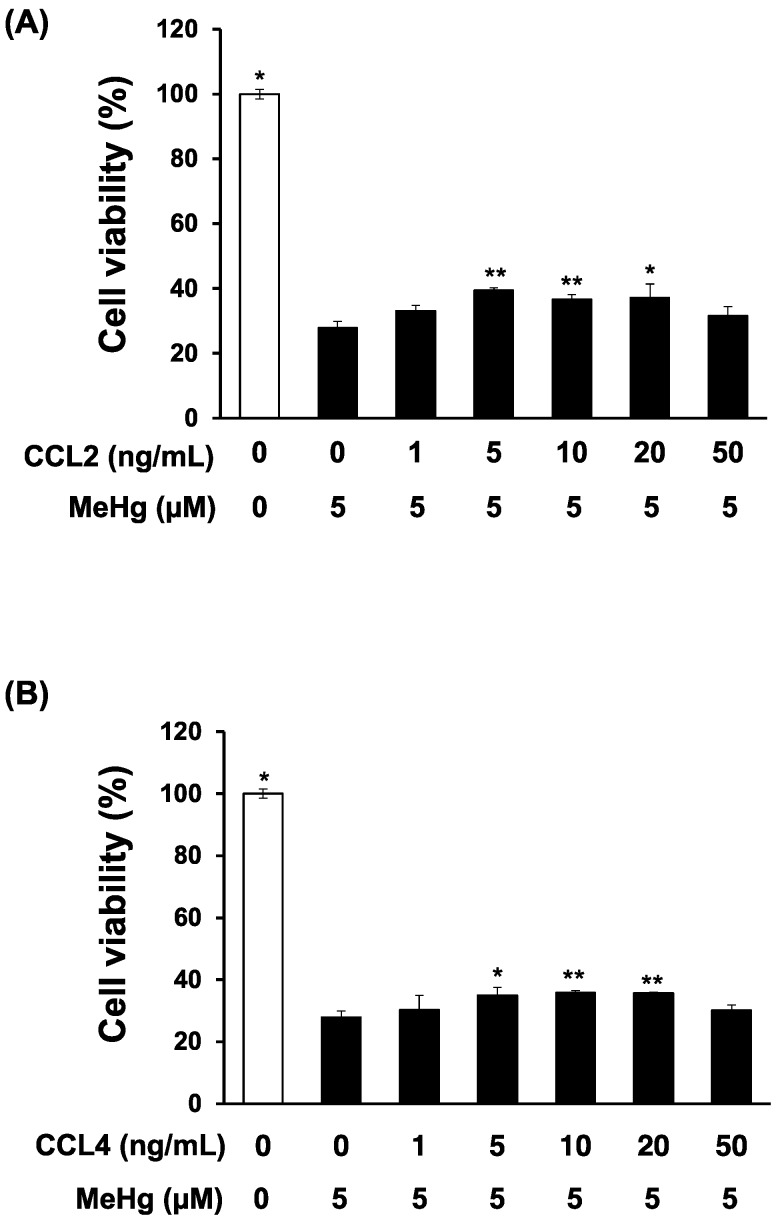
Effects of recombinant CCL2 or CCL4 on methylmercury-induced cytotoxicity in mouse primary cerebellar granule cells. Mouse primary cerebellar granule cells (neurons) (1 × 10^6^ cells/mL) were cultured in Neurobasal A medium containing 2% B25, 1% FBS, and 25 mM KCl in 12-well plates for 2 weeks. Recombinant CCL2 (**A**) or CCL4 (**B**) was then added to the culture medium, and 1 h later cells were exposed to 5 μM methylmercuric chloride (MeHg) for 24 h. Cell viability was measured by the alamarBlue^®^ assay. Data are presented as the mean ± S.D. * *p* < 0.05; ** *p* < 0.01 compared with the “0 ng/mL recombinant chemokine, 5 µM MeHg group”.

Godefroy et al. reported that addition of recombinant CCL2 into the medium of primary rat neuron cultures attenuated MeHg toxicity [16]. Therefore, we used primary mouse neuron cultures to investigate the effect of chemokines on MeHg sensitivity. Addition of recombinant CCL2 to the culture medium significantly attenuated the MeHg toxicity on primary mouse neurons (Figure 2A). The addition of recombinant CCL4 also significantly attenuated MeHg toxicity, to a greater extent than CCL2, indicating that CCL4, like CCL2, is a protective factor against MeHg neurotoxicity (Figure 2B).

### 3.3. CCL4 Expression Is Induced Prior to MeHg-Induced Cytotoxicity in C17.2 Cells

**Figure 3:**
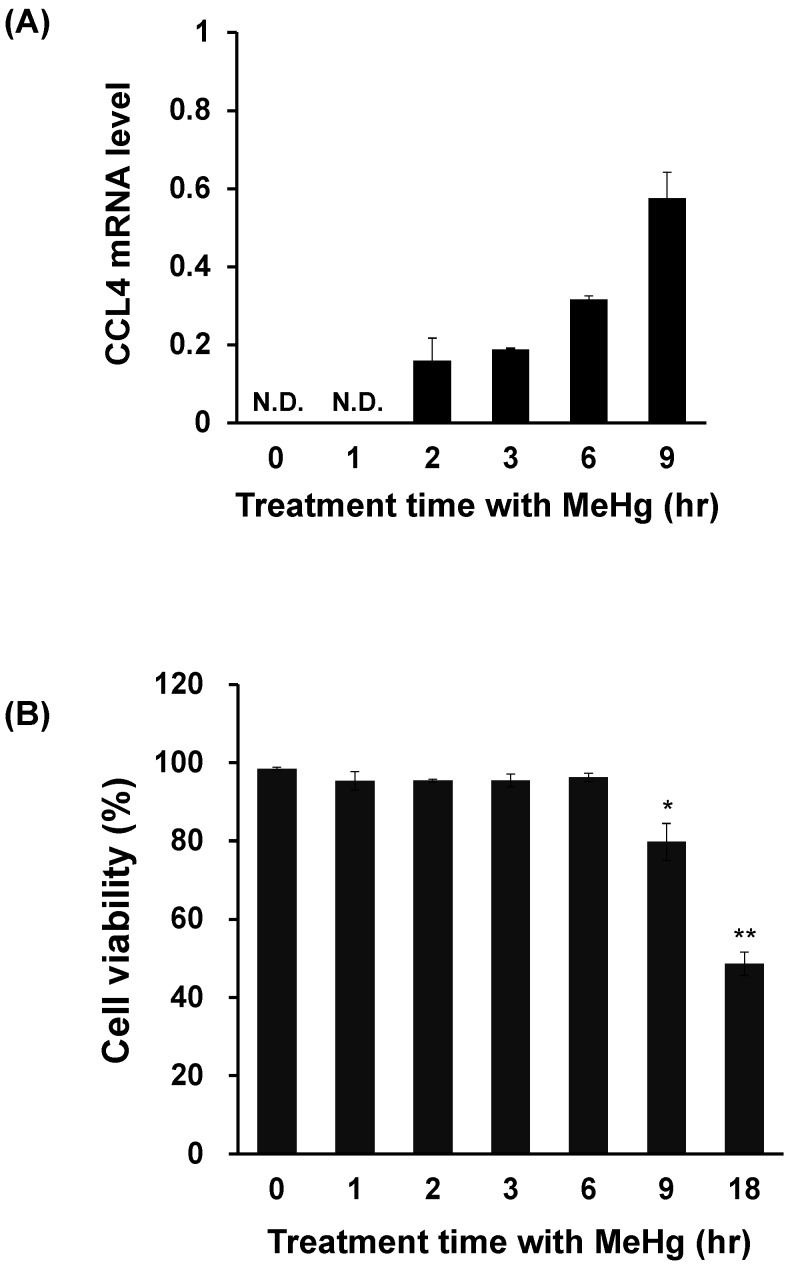
Relationship between cell viability and CCL4 expression in methylmercury-treated mouse C17.2 neural stem cells. C17.2 cells (4 × 10^5^ cells/2 mL) were seeded into 6-well plates. After incubation for 18 h, cells were treated with 10 μM methylmercuric chloride (MeHg) for the indicated times. (**A**) CCL4 mRNA levels were measured by quantitative real-time PCR. (**B**) Cell viability was determined by the trypan blue assay using the Vi-CELL cell counter. N.D.: not detected. Data are presented as the mean ± S.D. * *p* < 0.05; ** *p* < 0.01 compared with “0 h group”.

We investigated the effect of MeHg on CCL4 expression in C17.2 cells, a neural progenitor cell derived from the mouse brain. CCL4 expression was below detection threshold under normal conditions. However, increased CCL4 mRNA levels were detected from 2 h after MeHg treatment, and this increase continued over time up to 9 h (Figure 3A). However, when cell viability was measured by the trypan blue assay, cell viability decreased from 9 h after MeHg treatment (Figure 3B). This showed that even in C17.2 cells, CCL4 expression is induced prior to MeHg cytotoxicity.

### 3.4. Knockdown of CCL4 Enhances MeHg Cytotoxicity in C17.2 Cells

**Figure 4:**
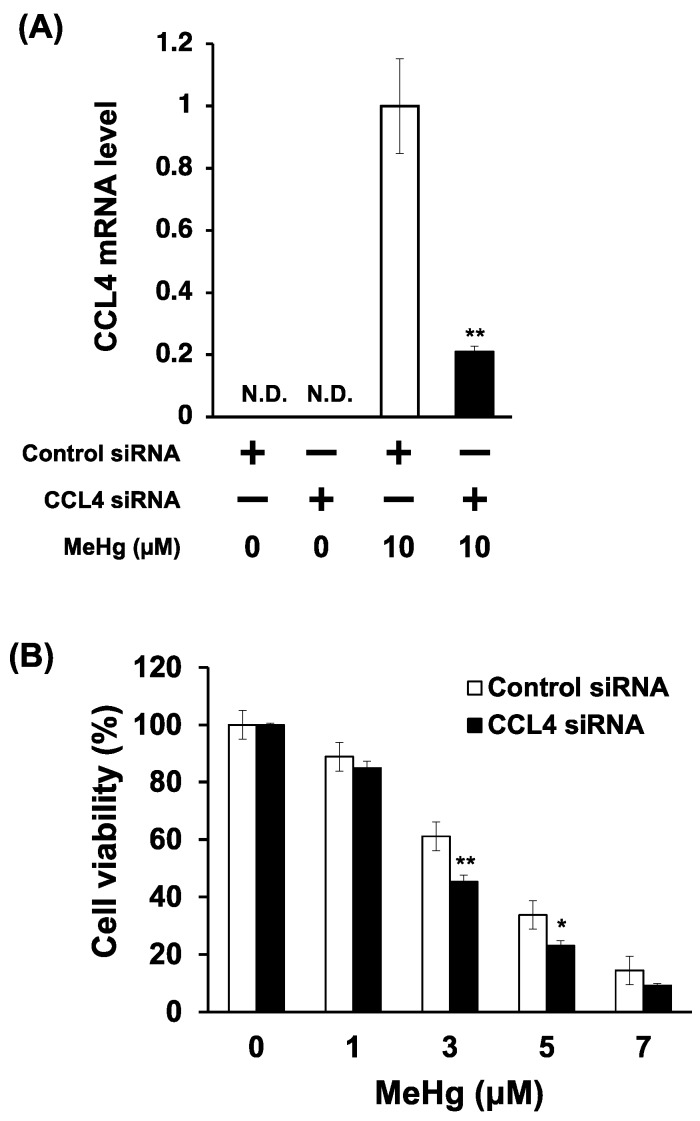
Effects of CCL4 knockdown on the sensitivity of C17.2 cells to methylmercury. C17.2 cells (1 × 10^4^ cells/90 μL) transfected with CCL4 siRNA were seeded into 96-well plates. After incubation for 18 h, transfected cells were treated with indicated concentrations of methylmercuric chloride (MeHg) for 24 h. (**A**) CCL4 mRNA levels were measured by quantitative real-time PCR. (**B**) Cell viability was measured by the alamarBlue^®^ assay. Data are presented as the mean ± S.D. N.D.: not detected. Statistical significance when compared to corresponding “control siRNA group” at each concentration point: * *p* < 0.05, ** *p* < 0.01.

To clarify the relationship between CCL4 expression and MeHg toxicity, we investigated the effects of CCL4 knockdown on the MeHg sensitivity of C17.2 cells. The introduction of siRNA to CCL4 reduced the induction of CCL4 expression by MeHg by about 60% (Figure 4A). CCL4 knockdown cells were more sensitive to MeHg than cells transfected with control siRNA (Figure 4B). This indicates that CCL4 has a protective role against MeHg toxicity.

## 4. Discussion

The present study showed that CCL4 expression in mouse-derived neuronal precursor C17.2 cells, as well as in the mouse brain, was induced prior to MeHg cytotoxicity. These findings indicate the possibility that the CCL4 expression induced by MeHg is not the result of cellular damage caused by MeHg, but a protective response to MeHg exposure. It is also known that the cell has a number of protective systems against toxicity of environmental toxicants and that those are activated in the early stage of exposure. Therefore, CCL4 induction in the early stage of MeHg exposure seems to be a protective action against its toxicity.

The transcription factor, NF-κB, is involved in the induction of cytokine expression [20,21]. CCL4 expression is induced in macrophages by lipopolysaccharide (LPS) and hydrogen peroxide and NF-κB is involved in this induction [22,23,24]. We recently found that knockdown of p65, a subunit of NF-κB, slightly suppressed the induction of CCL4 expression in response to MeHg in C17.2 cells (unpublished data). This suggests that while NF-κB is partially involved in the induction of CCL4 expression in response to MeHg, other transcription factors are also involved. It is possible that MeHg induces CCL4 expression due to the activation of transcription factors that differ from those activated by LPS and hydrogen peroxide. In future studies, we hope to clarify the mechanisms underlying brain-specific MeHg toxicity by determining how CCL4 is induced in response to MeHg.

In this study, CCL4 was newly identified as a protective factor against MeHg induced cytotoxicity. Recently, CCL2 was also identified as a protective factor against MeHg toxicity on primary rat neurons [16]. Although the protective mechanisms against MeHg toxicity through CCL2 and CCL4 are unknown, it is reported that expressions of CCL2 and CCL4 are increased early in the cerebral tissue of patients with posttraumatic brain contusions [25]. This suggests that both chemokines may play an important role as a defensive response in the brain injury.

It is known that CCL4 induces the production of cytokines including interleukin (IL)-1 and IL-6 [26,27,28]. Recently, Noguchi et al. reported that IL-6 expression is induced by MeHg, and that IL-6 has a protective role against MeHg-induced neurotoxicity [29]. It is thus thought that increased levels of CCL4, in response to MeHg, may enhance IL-6 production, thereby reducing MeHg toxicity. However, we could not confirm the induction of IL-6 expression in mouse brains treated with MeHg (data not shown). This suggests that IL-6 is not involved in reducing MeHg toxicity by CCL4.

MeHg induced higher CCL4 expression in the cerebellum compared with the cerebrum (Figure 1B). Nevertheless, loss of neurons was only observed in the cerebrum and was not confirmed in the cerebellum (Figure 1C). We have reported that tumor necrosis factor-α (TNF-α), an inflammatory cytokine, is selectively induced in the brain of mice treated with MeHg, and that the degree of induction was larger in the cerebrum than in the cerebellum [8]. In addition, TNF-α may enhance MeHg toxicity because a TNF-α antagonist attenuated MeHg toxicity for C17.2 cells [8]. Based on these findings, the degree of induction of CCL4 expression, which reduces MeHg toxicity, was higher in the cerebellum than in the cerebrum, and the degree of induction of TNF-α expression, which enhances MeHg toxicity, was low in the cerebellum. Therefore, MeHg toxicity may not be observed in the cerebellum under our experimental conditions. In addition, we found that the expression of CCL3 and of IL-19 are also specifically induced in the brain by MeHg [9,30]. Therefore, there are many cytokines that are specifically induced in the mouse brain by MeHg, and the combined action of these may play an important role in neuronal damage caused by MeHg. In future studies, we hope to clarify crosstalk between cytokine molecules that are specifically induced in the brain, which will help to elucidate the mechanisms involved in brain-specific MeHg toxicity.
